# Some Essentials in Diagnosing Abdominal Cases

**Published:** 1921-06-11

**Authors:** 


### ALIMENTARY INVESTIGATION.

### Some Essentials in Diagnosing Abdominal Cases.

In most cases alimentary disorders do not appear until the end of the third decade of life. Sir Arbuthnot Lane teaches that many of them owe their origin to continual and prolonged intestinal delay, and that the resistance of the patient is undermined by the toxæmia resulting from this delay. Occupation and personal habits all influence the condition of the alimentary mechanism, and these factors must be considered when estimating the nature and extent of any alimentary disorder. In any such investigation the most important question to decide is whether the lesion is malignant or not, and in order to form a reliable opinion no useful means of examination should be neglected. In making a diagnosis the careful consideration of general principles is of very great help, and often makes detailed and scientific investigations of confirmatory rather than of diagnostic importance.

### Simple Ulceration *v.* Neoplasm.

In this respect we consider the age of the patient. Alimentary neoplasms are encountered at an age nearer to fifty than to thirty years, whereas ulceration—stomach or duodenum—is mostly found in the younger group of patients. As regards the general nutrition of the patient, a marked wasting accompanies a malignant neoplasm, whereas in simple ulceration there is a small amount of anæmia and a certain amount of wasting due to inanition. The blood in a malignant case shows a relative lymphocytosis at the expense of the polymorphonuclear leucocytes. The inanition of the malignant case is due to a lack of desire for food, but in the case of simple ulceration the wasting is a result of the fear of eating food, because of the pain which always follows its ingestion.

Certain points in the history of these alimentary cases stand out, and by their prominence impress themselves on the mind of the patient: some of these are points of useful information, the two best being information concerning pain and blood. Pain, returning regularly and always bearing the same true relation to food, and in some cases being relieved by the taking of a small quantity of food, is mostly found in cases of ulceration. The appearance of vomited blood or a large quantity of altered blood in the fæces is also indicative of ulceration rather than malignant neoplasm.

So far, therefore, we have found more positive points of evidence for the diagnosis of ulceration rather than malignant disease; but it is in the detailed scientific methods of investigations that we can find the best assistance in establishing a diagnosis of malignancy. The examination of a test-meal affords very useful information concerning the condition of the stomach, and we have already referred in these pages to the new "fractional method." Hyperchlorhydria usually indicates ulceration, and hypochlorhydria is found in many cases of malignant disease. Another very useful channel is the aid of a spectroscopic examination of the fæces. This examination is to detect the continual presence of small quantities of occult blood: if present, there is very much in favour of a malignant neoplasm somewhere above the ileum, and the common site for these lesions is in the stomach or duodenum. Again, an investigation of the alimentary tract would not be complete without the use of *x*-rays and the opaque meal. It is needless to impress the number of instances in which malignant neoplasm and ulcerated areas have been detected by this method. Other lesions, too, are revealed by the *x*-ray. The different causes of "ileal delay," peritoneal adhesions, kinks, and wrongly placed appendices all come into this class, and the works of Sir Arbuthnot Lane and his colleagues have demonstrated the enormous value of this means of investigation.

### Direct Vision Instruments.

Finally, if the suspected lesion is close to either end of the alimentary canal it can be examined by direct vision. Œsophagoscopy has often revealed an early epithelioma, and sigmoidoscopy has often demonstrated the nature of an ulceration suspected to lie in the lower part of the large intestine.

Thus it may be justifiably suggested that the diagnosis of alimentary lesions is not very difficult, if a definite routine of investigation is followed and careful attention is paid to the information so gained. An early diagnosis is essential, for the most success attends the treatment of cases diagnosed early. Medical measures are usually sufficient for early cases of ulceration, and surgical treatment holds out the best hope of cure in cases both of chronic ulceration and of malignant disease. The great secret—and it cannot be too often urged—of the treatment of alimentary lesions is diagnosis at the earliest possible moment, using every known aid to diagnosis, and radical and energetic treatment by medical or surgical means according to the nature of the case.

